# Direct Observation of Anisotropic Coulomb Interaction in a Topological Nodal Line Semimetal

**DOI:** 10.1002/advs.202407437

**Published:** 2025-01-07

**Authors:** Hyo Won Kim, Junseo Jung, Gahee Lee, Taesu Park, Won‐Jun Jang, Hoil Kim, Jun Sung Kim, Ji Hoon Shim, Bohm‐Jung Yang, Sangjun Jeon

**Affiliations:** ^1^ Samsung Advanced Institute of Technology Suwon 13595 Republic of Korea; ^2^ Department of Physics and Astronomy Seoul National University Seoul 08826 Republic of Korea; ^3^ Center for Theoretical Physics (CTP) Seoul National University Seoul 08826 Republic of Korea; ^4^ Institute of Applied Physics Seoul National University Seoul 08826 Republic of Korea; ^5^ Department of Physics Chung‐Ang University Seoul 06974 Republic of Korea; ^6^ Department of Chemistry Pohang University of Science and Technology Pohang 37673 Republic of Korea; ^7^ Department of Physics Pohang University of Science and Technology Pohang 37673 Republic of Korea; ^8^ Center for Artificial Low Dimensional Electronic Systems Institute for Basic Science (IBS) Pohang 37673 Republic of Korea

**Keywords:** anisotropic coulomb screening, scanning tunneling microscopy, tilted dirac cone, topological nodal line semimetal

## Abstract

The fundamental characteristics of collective interactions in topological band structures can be revealed by the exploration of charge screening in topological materials. In particular, distinct anisotropic screening behaviors are predicted to occur in Dirac nodal line semimetals (DNLSMs) due to their peculiar anisotropic low‐energy dispersion. Despite the recent extensive theoretical research, experimental observations of exotic charge screening in DNLSMs remain elusive, which is partly attributed to the coexisting trivial bands near the Fermi energy. This study reports the first direct observation of highly anisotropic charge‐screening behavior in the DNLSM SrAs_3_. Through atomically resolved conductance measurements, a highly anisotropic charge‐screening pattern around charged impurities on a surface is demonstrated. Moreover, the combination of model studies and first‐principles calculations reveals the unique nature of the screening anisotropy in DNLSMs. The results of this study are expected to pave the way for understanding the profound collective behavior of interacting low‐energy fermions in topological materials.

## Graphical abstract

This research presents the first experimental evidence of unique charge screening in a Dirac nodal line semimetal. Using scanning tunneling microscopy and spectroscopy, the distinct direction‐dependent and concave‐shaped charge screening pattern around charged impurities on the surface of SrAs3 is directly observed. The unique nature of this anisotropic screening is revealed by combining these measurements with theoretical models and calculations.

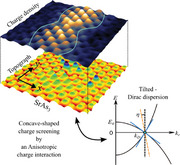

## 1 Introduction

Charge screening in metals is a collective phenomenon of many electrons that is associated with the polarizability of the electrons around the Fermi surface. The response of electrons to an external perturbation results in the formation of induced charges, which screen the impurity potential and restore charge neutrality. In a conventional metal, the screened long‐range Coulomb interaction is essentially reduced to a short‐range interaction, rendering the electron‐electron interaction negligible at the low‐energy limit. However, in the case of Dirac semi‐metals, wherein the electrons at the Fermi energy exhibit a relativistic linear dispersion with a vanishing density of states (DOS), the long‐range Coulomb interaction is marginally irrelevant.^[^ ^1^, ^2^, ^3^, ^4^ ^]^ Consequently, the electron scattering owing to logarithmically screened charged impurities is significantly different from that in nonrelativistic materials.^[^ ^5^ ^]^ Moreover, at the quantum critical point of a topological phase transition, where two Dirac points merge at a particular momentum, the low‐energy band structure develops anisotropic electronic dispersions that induce anisotropic charge polarization, thus resulting in anomalous low‐energy behaviors.^[^ ^6^, ^7^, ^8^ ^]^ This clearly demonstrates that the anisotropic low‐energy dispersion of topological semimetals is a promising method for investigating interaction‐induced novel low‐energy physics. However, despite the reports of enhanced many‐body effects in selected Dirac systems,^[^ ^9^, ^10^, ^11^, ^12^ ^]^ direct experimental observations of unconventional charge screening remain an unresolved issue. In particular, the observation of anomalous charge screening induced by anisotropic dispersion at the critical point requires fine‐tuning of material parameters, which is practically challenging to achieve in real materials.^[^ ^13^ ^]^ Although this phenomenon can be observed in specific photonic systems,^[^ ^14^, ^15^ ^]^ it is crucial to find alternative systems that inherently host anisotropic low‐energy band structures for its observation.

Dirac nodal line semimetals (DNLSMs) are topological materials that exhibit symmetry‐ensured 1D band crossings in the Brillouin zone near the Fermi energy.^[^ ^16^, ^17^, ^18^ ^]^ The band dispersion around the touching energy is linear along the directions perpendicular to the nodal line, whereas it is nondispersive or quadratic along the nodal line, a quasiparticle behavior known as semi‐Dirac fermions.^[^ ^19^ ^]^ Owing to their unique electronic structures, DNLSMs exhibit a nontrivial Berry phase and drumhead surface states.^[^ ^13^, ^20^, ^21^, ^22^ ^]^ Unique charge screening and long‐range Coulomb interactions have been predicted,^[^ ^23^, ^24^, ^25^, ^26^, ^27^ ^]^ and a strong electronic correlation has been observed, as evidenced by a significant reduction in the free‐carrier Drude weight and unconventional mass enhancement.^[^ ^28^, ^29^ ^]^

This study investigated unusual charge screening in SrAs_3_, which is a DNLSM material,^[^ ^30^, ^31^ ^]^ by observing the impurity‐induced anisotropic charge distributions through scanning tunneling microscopy (STM) and spectroscopy (STS).^[^ ^32^ ^]^ The presence of an isolated nodal ring in SrAs_3_ facilitates the unambiguous identification of the signature of non‐trivial charge screening.^[^ ^33^, ^34^ ^]^ We directly visualized the anisotropic and long‐range charge screening around the lattice deficiency experimentally. Our model calculations, in conjunction with density functional theory (DFT) calculations, demonstrated that the observed anisotropic charge screening is a consequence of the tilted Dirac dispersion within the nodal ring of SrAs_3_. Moreover, we explored the electric field‐induced charge filling in SrAs_3_, which provided additional insights into charge screening across the band inversion energy, thereby distinguishing the charge correlations in both topological bands within the band inversion energy and trivial bands in the non‐inverted energy region.

## 2 Band Anisotropy of DNLSMs

**Figure 1:**
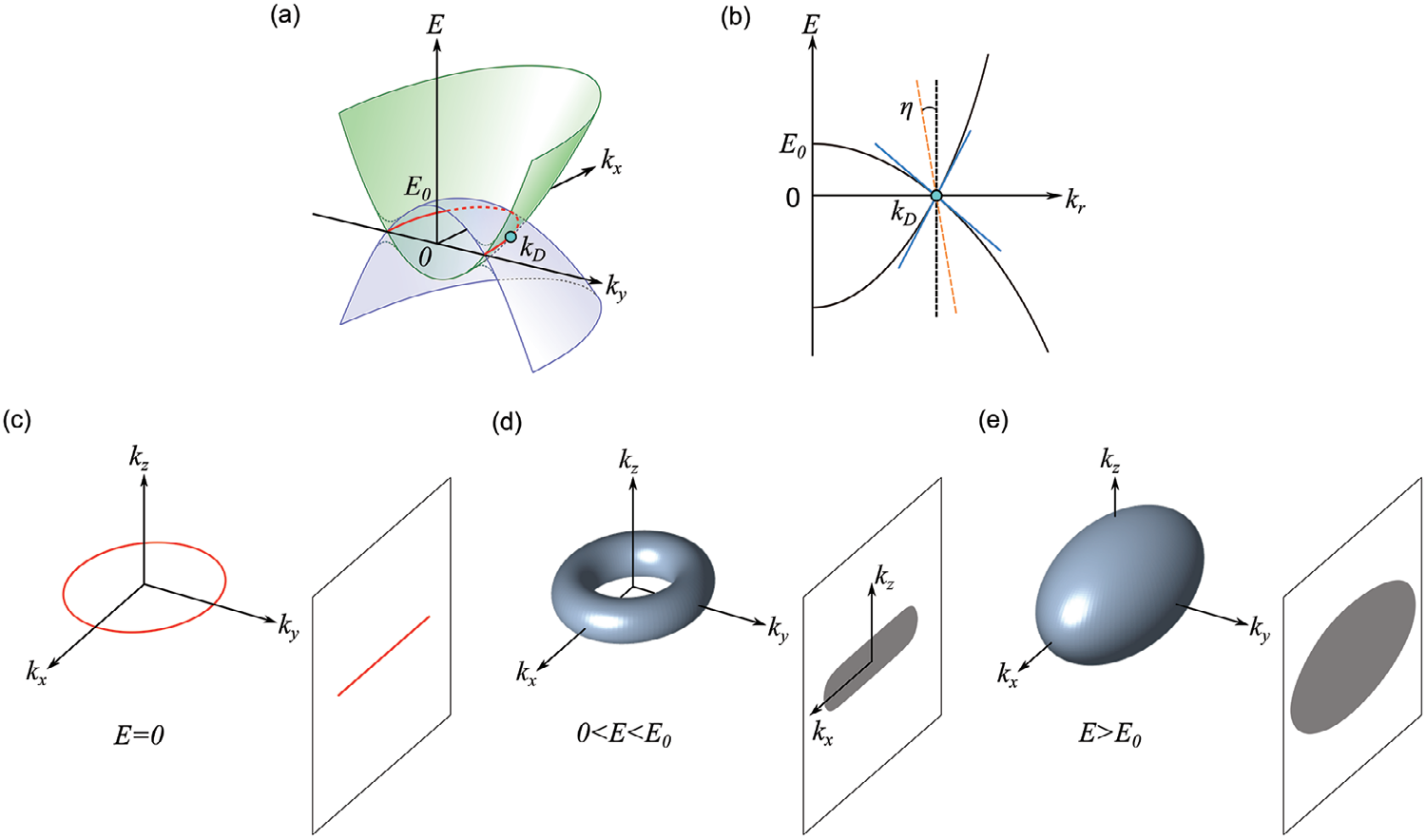
a) Schematic of *k_x_* and *k_y_* dispersion around the band inversion point. The red ring is the topologically protected nodal ring lying in a *k_x_* − *k_y_* plane. The band inversion energy is denoted as *E*_0_. b) The anisotropic Dirac dispersion at a point (*k_D_*) on the nodal line. The tilting angle is denoted as η. c–e) Shape of Fermi surfaces and their projected 2D Fermi surfaces normal to the *k_y_* axis for *E*  =  0, 0 < |*E*| < *E*_0_, and |*E*| > *E*_0_, respectively.

Within a DNLSM, the parabolic conduction and valence bands overlap, resulting in a distinct ring‐shaped contour composed of topologically protected crossing points (red curve in **Figure** **1**a) The different effective masses of these two bands generally result in a tilted Dirac dispersion at each point along the nodal ring. The cross‐sectional tilted Dirac dispersion at Momentum *k_D_* is shown in Figure 1b. The degree of the tilted angle (η) determines the anisotropy of the Dirac velocity, which varies based on the direction of dispersion. The 1D‐like Fermi surface (Figure 1c) when the chemical potential crosses the Dirac nodal line at *E =* 0, evolves into a torus‐shaped Fermi surface (Figure 1d) when this potential lies below the band inversion energy (*E*_0_), that is, 0 < *E* < *E*_0_. When the charge filling reaches a level at which the chemical potential overcomes *E*_0_ (*E* > *E*_0_), the Fermi surface transitions into an ellipsoidal form. Consequently, the conduction electrons lose their topological characteristics and behave as trivial electrons under electrical perturbations (Figure 1e). The projection of the bulk Fermi surface onto a plane perpendicular to the plane of the nodal ring, which is the plane investigated in this study, resulted in a markedly anisotropic two‐dimensional projected Fermi surface, particularly when |*E*| < |*E*_0_|.

## 3 Crystal and Electronic Structure of SrAs_3_

**Figure 2:**
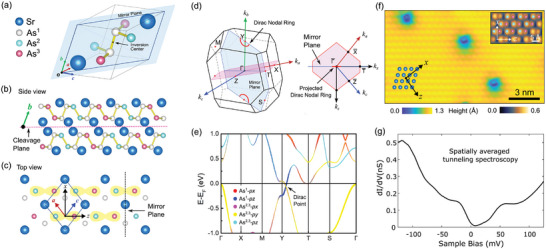
a) A conventional unit cell structure of SrAs_3_. The mirror plane (blue‐colored plane) and the inversion center are marked. b,c) Side (b) and top (c) views of the crystal structure of SrAs_3_. The puckered As and Sr layers stack alternately along the *b*‐axis. The natural cleavage plane lies between Sr layers (marked as the purple dashed line in the side view), which is perpendicular to the mirror plane. d) 3D BZ of the SrAs_3_ and the projected BZ along the *b*‐axis. The Dirac nodal ring is located in the mirror‐invariant plane around the Y points. e) Electronic band structure of SrAs_3_ from density functional calculations with modified Becke–Johson exchange potential. The colors on the lines indicate the orbital characters. Spin‐orbit coupling effect is not included in the calculation. f) A typical STM image of a cleaved surface with a few atomic deficiencies (*V* = 1 V and *I* = 100 pA). The inset shows a low‐bias topography that exhibits As pairs (*V* = −0.2 V and *I* = 30 pA). The crystalline direction *x* (parallel to the mirror plane) and *z* (normal to the mirror plane) are indicated. g) Spatially averaged scanning tunneling spectroscopy (STS) on a cleaved surface of SrAs_3_ (*V*_set_ = −120 mV, *I* = 100 pA, *V*_osc_ = 10 mV).

SrAs_3_ is a layered compound with a puckered polyanionic structure containing As‐As dimers. The symmetry group is C2/m, which preserves the inversion and mirror symmetries, as illustrated in **Figure** **2**a. In the primitive cell, strontium and arsenic atoms (As^1^) were located on the mirror plane (blue plane in Figure 2a), and the other two arsenic atoms, As^2^ and As^3^, were located symmetrically with respect to the mirror plane. First‐principles calculations based on the generalized gradient approximation (GGA) approximation predicted the presence of a nodal ring around the Y point in the Brillouin zone (Figure 2d,e), which is protected by mirror symmetry as well as the combination of time‐reversal *T* and inversion symmetry *P* when spin‐orbit coupling (SOC) is neglected.^[^ ^30^ ^]^

Although the initial first‐principles calculations showed a coexisting trivial band that crosses the Fermi energy,^[^ ^30^ ^]^ recent quantum oscillation^[^ ^33^ ^]^ and ARPES measurements^[^ ^21^, ^34^ ^]^ have shown that the Dirac nodal ring in SrAs_3_ is isolated from other trivial bands. The DFT calculations using modified Becke–Johnson (mBJ) potential captured the experimentally observed band structure of SrAs_3_ (Figure 2e) whose electron‐like dispersion around the T point was found to have shifted above the Fermi energy and hole‐like band around Г point that shifted below the Fermi energy. Consequently, the transport properties of SrAs_3_ were fully characterized by electrons in the Dirac nodal ring.^[^ ^33^ ^]^ Spin‐orbit coupling (SOC) is predicted to open a small gap at the crossing lines that may convert SrAs_3_ into a topological insulator or Dirac semimetal.^[^ ^30^ ^]^ However, recent ARPES measurements show that the gap is minor within the energy resolution of measurements.^[^ ^34^ ^]^ Moreover, even with a gap at the crossing points, as reported in the optical conductivity measurements,^[^ ^35^ ^]^ the tilted nature of the DNL in SrAs_3_ preserves its semi‐metallic behavior^[^ ^33^ ^]^ (see Section I, Supporting Information).

The cleaved surface, as measured by STM, exhibited a rhombic lattice with a few defects (Figure 2f). Because the puckered As layers were located between the Sr layers, the natural cleavage plane was between the Sr layers (purple dashed line in Figure 2b). Notably, the topographic features were influenced by the measurement energy because of the energy‐dependent spectral weights of Sr and As atoms (Figure 2e; Section II, Supporting Information). Depending on the measurement energy, the lattices of Sr atoms (Figure 2f) and pairs of As atoms (inset of Figure 2f) are selectively imaged. The averaged LDOS in a defect‐free region (Figure 2g) exhibited a slight suppression of the DOS near the Fermi energy, which matched the isolated Dirac nodal ring reported in the quantum transport of SrAs_3_ and DFT calculation.^[^ ^33^ ^]^ However, because the Dirac energy varies with momentum in SrAs_3_, observing a finite DOS around the Fermi energy does not rule out the possibility of a finite SOC gap.^[^ ^34^ ^]^ In regions with As deficiencies, a gap‐like DOS near the Fermi level is observed, but this is limited to the local region around the deficiency site (see Section I, Supporting Information). The cleaved surface disrupted the mirror symmetry of SrAs_3_; therefore, As^2^ and As^3^ revealed distinct height profiles in the high‐resolution STM topography (further details in Section III, Supporting Information). Our DFT calculations showed that the surface energy of SrAs_3_ could be lowered by breaking the mirror symmetry. Furthermore, the spectral weight calculation captured subtle differences in the local density of states (LDOS) between As^2^ and As^3^ (Section IV, Supporting Information).

## 4 Induced Charge Distribution by an Impurity

**Figure 3:**
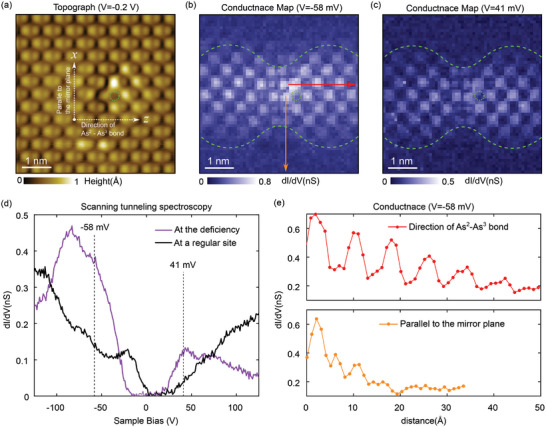
a) STM topographic data with an As atom deficiency (the green dotted ring) (*V* = −200 mV, *I* = 30 pA). b,c) dI/dV maps measured for *V* = −58 mV b), *V* = 41 mV c) at the same area as (a). The conductance density enhancement owing to the As vacancy is observed (the green dashed lines indicate the shape of the charge distribution). d) dI/dV spectrum taken at the As vacancy site and a regular As site 5 nm from the vacancy site. The measurement energy of (b), (c) are marked. e) The conductance profiles along the red and orange arrows in (b).

Atomic impurities on the surface of SrAs_3_ result in an unconventional alteration of the lattice structure and charge distribution. Arsenic deficiency was the most prevalent impurity, which added hole carriers to the system (dashed circles in **Figure** **3**a). The positions of the As^2^–As^3^ pairs deviated strongly near the impurity site from the expected symmetric positions of the bulk crystalline structure. Notably, the positional changes from the pristine lattice extended more than 5 nm away from the deficiency site along the As^2^─As^3^ bond, which is normal to the mirror plane. Meanwhile, the change extended by ≈1 nm along the direction of the mirror plane (see the topographic profiles in Section III, Supporting Information).

The charge distribution owing to the presence of atomic deficiency is highlighted in the constant energy conductance map. Figure 3b,c show the spatial variation of the conductance measured at the sample biases of −58 and 41 mV, respectively, relative to the Fermi energy at the same location as in Figure 3a. This revealed the checkerboard‐like conductance enhancement and the butterfly‐shaped contour (the dashed green lines) surrounding the deficiency site. Moreover, the mirror symmetry between As^2^ and As^3^ was strongly broken, and the orientation of the concave feature was aligned with the projection of the mirror plane on the cleaved surface. Due to the broken mirror symmetry on a surface, the geometric center of the conductance enhancement shifts by one atomic site from the As deficiency (see Section III, Supporting Information).

In Figure 3d, the dI/dV spectrum acquired at the As‐deficiency site was compared with that obtained at a regular As site located away from the deficiency site. At the deficiency site, the impurity‐induced electric potential coupled with the electric field generated by the STM tip resulted in a shift in the central position of the conductance suppression in energy.^[^ ^36^ ^]^ The broad enhancement of conductance observed at both negative bias (−70 mV) and positive bias (40 mV) was attributable to the impurity‐induced states. Additional charges introduced by defects facilitated a local alteration of the electric potential and the accumulation of screening charges, which are governed by the torus‐shaped Fermi surface of SrAs_3_.

Notably, the impurity‐induced conductance exhibited an extremely slow decay with anisotropic length scales, as shown in Figure 3e. This pronounced direction‐dependent conductance modification, which indicates a highly anisotropic distribution of the induced charge, cannot be explained by the typical exponential decay associated with Thomas–Fermi screening. The decay rate in the vertical direction appeared slightly slower than the predicted 1/*r* ^2^ decay for the DNLSM at intermediate distances.^[^ ^27^ ^]^ Moreover, the decay rate along the As bonding direction was lower than the expected 1/*r* behavior at short distances^[^ ^27^ ^]^ (Figure S5, Section III, Supporting Information). To fully account for this unusual charge behavior, a more realistic model beyond the simplified DNLSM model that incorporates the finite Fermi energy away from the Dirac crossing along with a tilted Dirac dispersion must be considered.

## 5 Anisotropic Charge Screening in DNLSMs

**Figure 4:**
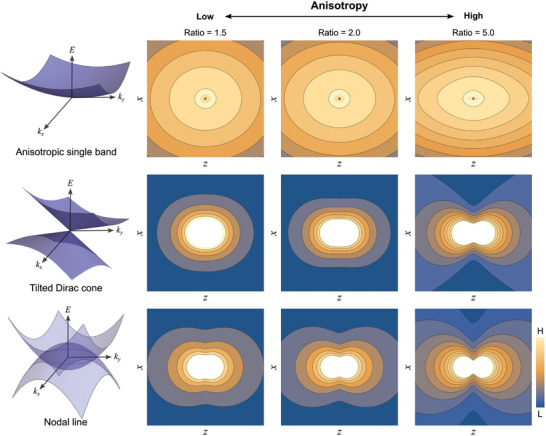
Anisotropic charge distribution for an anisotropic single band, a tilted Dirac cone, and a nodal line. Screening charge distribution on the surface of each system for different anisotropy ratios. The horizontal axis (*z*) and vertical axis (*x*) of the charge maps align with the directions of *k_x_* and $k_{z}$, respectively. The anisotropy ratio of a single band is defined as *R* = *m_z_*/*m_x_*, which is the mass ratio between *k_x_* and *k_z_* direction. The anisotropy ratio of a tilted Dirac cone and a nodal line is defined as a combination of the velocity anisotropy and the tilt expressed as *R* = (1 + η^2^)γ^2^. The velocity anisotropy and tilt of the nodal line are calculated from the average velocities of the nodal line. The screening charge of an anisotropic single‐band model does not form a butterfly shape. Both the tilted Dirac cone and nodal line display butterfly‐shaped charge configurations; however, the nodal line exhibits a concave shape even at a relatively small anisotropy ratio.

To understand the unconventional charge screening in SrAs_3_, we constructed a two‐band effective Hamiltonian that characterizes the DNLSMs. During the formulation of the low‐energy continuum theory, we considered the specific symmetries of SrAs_3_ and determined the Hamiltonian parameters using DFT calculations with the mBJ functional approach, as outlined in Section V (Supporting Information). The model Hamiltonian contained anisotropic Dirac velocities along two distinct directions: the radial direction (*r*) lying in the mirror plane, and the normal direction (*z*), which is perpendicular to the mirror plane (direction of As^2^─As^3^ bonding), exhibiting linear dispersion. By contrast, the direction tangential to the nodal ring exhibited a quadratic dispersion. Furthermore, the Dirac dispersion at the nodal points is tilted toward the center of the nodal ring, as depicted in Figure 1b. The following Hamiltonian captures the tilted Dirac dispersion at a point on the nodal line.

(1) $$H\left(k\right)=v_{r0}k_{r}\sigma_{0}+v_{r}k_{r}\sigma_{1}+v_{z}k_{z}\sigma_{3}=v_{r}\left(\eta k_{r}\sigma_{0}+k_{r}\sigma_{1}+\frac{1}{\gamma }k_{z}\sigma_{3}\right)$$

where *k_r_* (*k_z_*) and *v_r_* (*v_z_*) are the momenta and Dirac velocities along the *r* and *z* directions, respectively, and σ_*i*_’s are the Pauli matrices. The parameters defined as $\eta =\frac{v_{r0}}{v_{r}}$ and $\gamma =\frac{v_{r}}{v_{z}}$ indicate the tilting strength and velocity anisotropy of the Dirac cone, respectively. The specific values of the parameters obtained from the DFT calculations are summarized in Figure S9 (Supporting Information) Section V. The static polarization function from the tilted Dirac Hamiltonian is:

(2) $$Re\Pi \left(q,\omega =0\right)=-C\sqrt{\frac{q_{r}^{2}+\frac{q_{z}^{2}}{\gamma^{2}}}{1-\eta^{2}\mathrm{co}s^{2}\phi }}$$

where $C=\frac{g}{16\hbar v_{r}v_{z}}$, $\cos\phi =\frac{q_{r}}{\sqrt{q_{r}^{2}+\frac{q_{z}^{2}}{\gamma^{2}}}}$, and *g* represents the spin degeneracy. As the static polarization determines the screening charge distribution ρ(*q*) in momentum space induced by a charged impurity, the real space charge distribution can be obtained via the Fourier transformation of the polarization function. The anisotropy and shape of the charge screening are entirely defined by the γ and η factors for a tilted Dirac cone. As shown in **Figure** **4**, the simulated charge distribution was subjected to a transition from a convex to a concave shape with an increase in the discrepancy between the Dirac velocities (γ value) and tilting strength (η value).

The individual polarization functions at each nodal point were summed to obtain the total charge distribution around the Dirac nodal ring. The explicit analytical form of the total polarization function of the nodal line is presented in Section V (Supporting Information). Similar to the case of a single tilted Dirac cone, the combination of anisotropic Dirac velocities (*v_r_* > *v_z_*) and an axis tilted toward the center of the nodal ring drove the charge distribution contour into a concave shape in SrAs_3_.

To further demonstrate that the butterfly shape of the screened charge is a characteristic feature only observable in the NLSM or highly anisotropic Dirac cones, we consider the static polarization function of a single quadratic band with an anisotropic mass represented by the Hamiltonian $H\left(k\right)=\frac{k_{x}^{2}}{2m_{x}}+\frac{k_{y}^{2}}{2m_{y}}+\frac{k_{z}^{2}}{2m_{z}}$ where *m*_*x*,*y*, *z*_ indicate the effective masses along the three directions. The corresponding static polarization function is expressed as:

(3) $$Re\Pi^{1band}\left(q,\omega =0\right)=\frac{mk_{F}}{4\pi^{2}}\left(1+\left(\frac{1}{2x}-\frac{x}{2}\right)\ln\left(\frac{1+x}{1-x}\right)\right)$$

where *E_F_* is the Fermi energy, $k_{F}=\sqrt{2{\left(m_{x}m_{y}m_{z}\right)}^{\frac{1}{3}}E_{F}},$ $x=\frac{\tilde{q}}{2k_{F}}$, and $\tilde{q}=\sqrt{{\left(m_{x}m_{y}m_{z}\right)}^{\frac{1}{3}}\left(\frac{q_{x}^{2}}{m_{x}}+\frac{q_{y}^{2}}{m_{y}}+\frac{q_{z}^{2}}{m_{z}}\right).}$ In this polarization function, regardless of the Fermi energy and the mass anisotropy, the induced charge density ρ_*ind*_(*q*) does not form a butterfly shape. For appropriate demonstration, the relevant screening charge distribution is plotted in the top panels of Figure 4, wherein the mass tensors set along the radial directions are the same *m_x_* = *m_y_*; the mass ratio between the radial direction *m_x_* and normal direction *m_z_* was varied. As evident, regardless of the mass ratio, $\frac{m_{z}}{m_{x}}$, the contour did not exhibit a butterfly shape.

## 6 Topological Crossover by the Tip‐Induced Band Bending

When a material with a low DOS is placed in proximity to a metallic tip, the localized electric field emanating from the tip can facilitate the accumulation or depletion of the electronic charge density owing to the local shift of the energy bands. This phenomenon, known as tip‐induced band bending (TIBB), is challenging when interpreting STM measurements. The TIBB is influenced by various factors, including the distance between the tip and sample, the shape of the tip, the electrical properties of the material, and the voltage applied between the tip and sample.^[^ ^37^ ^]^ Despite these challenges, TIBB can locally gate the surface under the tip, offering the opportunity to observe the collective behavior of electrons by modifying their chemical potential.^[^ ^36^ ^]^

**Figure 5:**
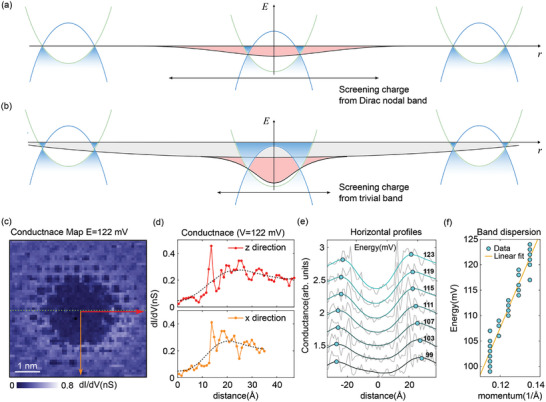
a) Charge distribution without tip‐induced band bending (TIBB). The inverted band structure and the position‐dependent chemical potentials are overlaid on the horizontal axis (*r*), which represents the distance from the impurity. Impurity potential represented by the red‐shaded region is screened by the Dirac electrons. b) Strong TIBB (gray shaded region) with the impurity potential (red shaded region) can induce sufficient charges such that the local Fermi energy exceeds the band inversion energy *E*_0_. The impurity potential is then screened by electrons in a trivial band. c) Conductance map measured for *V* = 122 mV at the same area as in Figure 3a. d) Conductance profiles along the red (*z* direction) and orange (*x* direction) arrows in c). Dashed lines plot the local average of each profile. e) Energy‐dependent conductance profile (grey line) and a local average of each profile (cyan line) obtained along the dashed green line in (c). Cyan dots indicate the local maxima of Friedel oscillation. f) Band dispersion obtained from the wavelength of Friedel oscillation shown in (e). The linear fit exhibits *k_z_* = 0.11 ${Å}^{-1}$ and *k_x_* = 0.15 ${Å}^{-1}$, which perfectly matches with the DFT calculations. The observed Fermi velocities of *v_z_* ≈ *v_x_* ≈ 0.9 (ÅeV) at *V* = 100 mV is underestimated compared to the DFT calculations owing to the tip‐induced band bending.

In our measurements, the TIBB locally altered the shape of the Fermi surface and the topological properties of the electrons. Under low‐bias conditions, screening charges predominantly originate from the Dirac nodal ring. The TIBB effect in the DNLSM is schematically illustrated in **Figure** **5**a wherein the electric field introduced by the tip is modest, resulting in long‐range anisotropic charge accumulation around the impurity, as shown in Figure 3. However, when a sufficiently high bias was applied between the tip and sample, a significant accumulation of charges occurred beneath the tip, which resulted in the local Fermi energy exceeding the band‐inversion energy, as illustrated in Figure 5b. Our conductance map measured with a V_bias_ greater than 100 mV revealed a relatively isotropic LDOS distribution around the deficiency, as shown in Figure 5c,d. This resembled the Friedel charge oscillation pattern of topologically trivial conducting materials. The radii of the Fermi surface along the *z* and x directions were 0.11 and 0.15 ${Å}^{-1}$, which perfectly matched the DFT values of the electron‐like band above the band inversion energy. The radii of the Fermi surface increased with increasing measurement energy, as shown in Figure 5e,f. However, the difference between the energy measured by the TIBB and the effective energy underestimated the Fermi velocity in the STM measurements.

## 7 Conclusion

To conclude, we report the first direct visualization of the anisotropic charge screening effects induced by the interacting inverted topological band structure of a DNLSM. Although nontrivial charge screening is expected in various topological semimetals^[^ ^24^, ^27^ ^]^ and low‐dimensional materials,^[^ ^38^, ^39^ ^]^ the direct experimental observation of long‐range charge screening with a concave contour is highly unexpected results. The unique anisotropic charge distribution observed in the STM/STS measurements was attributed to the linear electronic dispersion, along with tilted Dirac cones, which advances our comprehension of collective electron interactions within topological band structures. We expect that this long‐ranged screening and intrinsic anisotropic behavior in electron interactions will potentially lead to strong directional variations in charge transport at low temperatures. Furthermore, the distinctive charge screening signatures observed under controlled charge levels are poised to provide a key advancement toward the complete characterization of the interacting band topology in correlated topological materials.

## 8 Experimental Section

Single‐crystal growth and characterization: SrAs_3_ single crystals were synthesized using the Bridgman method.^[^ ^33^, ^40^ ^]^ The grown single crystals had diameters of ≈12 mm. High crystallinity and stoichiometry were confirmed by X‐ray diffraction and energy‐dispersive spectroscopy.

STM/STS measurements: The experiments were performed in a commercial low‐temperature STM (UNISOKU Co., Ltd.). A SrAs_3_ single‐crystal sample was cleaved in an ultrahigh vacuum chamber (10^−10^ Torr) at 300 K and then transferred to the low‐temperature sample stage. The temperature was maintained at 2.7 K during the measurement. The STS were obtained employing a lock‐in technique with a bias modulation at 1 kHz.

Density functional calculations: The electronic structure of bulk SrAs_3_ was determined via first‐principles calculations using the full‐potential linearized augmented plane wave method implemented in the WIEN2K package.^[^ ^41^ ^]^ For DFT calculations, the crystal structure of bulk SrAs_3_ was obtained from reported X‐ray diffraction measurements.^[^ ^40^ ^]^ The modified Becke–Johnson (mBJ) exchange‐correlation function was employed to obtain an accurate description of the band structure of DNLSM SrAs_3_.^[^ ^42^, ^43^ ^]^ The detailed parameters to be used in the model Hamiltonian were extracted from elaborate band structure calculations along the radial or tangential directions to the Dirac points on the nodal ring.

## Conflict of Interest

The authors declare no conflict of interest.

## Supporting information



Supporting Information

## Data Availability

The data that support the findings of this study are available from the corresponding author upon reasonable request.

## References

[1] D. T. Son , Phys. Rev. B 2007, 75, 235423.

[2] V. N. Kotov , B. Uchoa , V. M. Pereira , F. Guinea , A. H. Castro Neto , Rev. Mod. Phys. 2012, 84, 1067.

[3] H. Isobe , N. Nagaosa , Phys. Rev. B 2012, 86, 165127.

[4] P. Goswami , S. Chakravarty , Phys. Rev. Lett. 2011, 107, 196803. 22181632 10.1103/PhysRevLett.107.196803

[5] R. R. Biswas , S. Sachdev , D. T. Son , Phys. Rev. B 2007, 76, 205122.

[6] B.‐J. Yang , E.‐G. Moon , H. Isobe , N. Nagaosa , Nat. Phys. 2014, 10, 774.

[7] H. Isobe , B.‐J. Yang , A. Chubukov , J. Schmalian , N. Nagaosa , Phys. Rev. Lett. 2016, 116, 076803. 26943551 10.1103/PhysRevLett.116.076803

[8] G. Y. Cho , E.‐G. Moon , Sci. Rep. 2016, 6, 19198. 26791803 10.1038/srep19198 PMC4726365

[9] L. A. Wray , S. Xu , Y. Xia , D. Hsieh , A. V. Fedorov , Y. S. Hor , R. J. Cava , A. Bansil , H. Lin , M. Z. Hasan , Nat. Phys. 2011, 7, 32.

[10] D. C. Elias , R. V. Gorbachev , A. S. Mayorov , S. V. Morozov , A. A. Zhukov , P. Blake , L. A. Ponomarenko , I. V. Grigorieva , K. S. Novoselov , F. Guinea , A. K. Geim , Nat. Phys. 2011, 7, 701.

[11] N. Xu , Z. W. Wang , A. Magrez , P. Bugnon , H. Berger , C. E. Matt , V. N. Strocov , N. C. Plumb , M. Radovic , E. Pomjakushina , K. Conder , J. H. Dil , J. Mesot , R. Yu , H. Ding , M. Shi , Phys. Rev. Lett. 2018, 121, 136401. 30312078 10.1103/PhysRevLett.121.136401

[12] S.‐H. Kang , S. Jeon , H.‐J. Kim , W. Ko , S. Cho , S. H. Kang , S. W. Kim , H. Yang , H. W. Kim , Y.‐W. Son , Phys. Rev. B 2022, 105, 045143.

[13] C. Fang , H. Weng , X. Dai , Z. Fang , Chinese Phys. B 2016, 25, 117106.

[14] M. Milićević , G. Montambaux , T. Ozawa , O. Jamadi , B. Real , I. Sagnes , A. Lemaître , L. L. Gratiet , A. Harouri , J. Bloch , A. Amo , Phys. Rev. 2019, 9, 031010. 10.1103/PhysRevLett.125.186601 33196264

[15] S. Hu , Z. Guo , W. Liu , S. Chen , H. Chen , Nat. Commun. 2024, 15, 2773. 38555373 10.1038/s41467-024-47125-7 PMC10981722

[16] A. A. Burkov , M. D. Hook , L. Balents , Phys. Rev. B 2011, 84, 235126.

[17] T. Bzdušek , Q. S. Wu , A. Rüegg , M. Sigrist , A. A. Soluyanov , Nature 2016, 538, 75. 27556949 10.1038/nature19099

[18] J. Y. Lin , N. C. Hu , Y. J. Chen , C. H. Lee , X. Zhang , Phys. Rev. B 2017, 96, 075438.

[19] Y. Shao , S. Moon , A. N. Rudenko , J. Wang , J. Herzog‐arbeitman , M. Ozerov , D. Graf , Z. Sun , R. Queiroz , S. H. Lee , Y. Zhu , Z. Mao , M. I. Katsnelson , B. A. Bernevig , D. Smirnov , A. J. Millis , D. N. Basov , Phys. Rev. X 2024, 14, 41057.

[20] M. Biderang , A. Leonhardt , N. Raghuvanshi , A. P. Schnyder , A. Akbari , Phys. Rev. B 2018, 98, 075115.

[21] M. M. Hosen , G. Dhakal , B. Wang , N. Poudel , K. Dimitri , F. Kabir , C. Sims , S. Regmi , K. Gofryk , D. Kaczorowski , A. Bansil , M. Neupane , Sci. Rep. 2020, 10, 2776. 32066748 10.1038/s41598-020-59200-2 PMC7026427

[22] Y. Yang , H. Xing , G. Tang , C. Hua , C. Yao , X. Yan , Y. Lu , J. Hu , Z. Mao , Y. Liu , Phys. Rev. B 2021, 103, 125160.

[23] Z. Yan , P.‐W. Huang , Z. Wang , Phys. Rev. B 2016, 93, 085138.

[24] Y. Huh , E.‐G. Moon , Y. B. Kim , Phys. Rev. B 2016, 93, 035138.

[25] W. B. Rui , Y. X. Zhao , A. P. Schnyder , Phys. Rev. B 2018, 97, 161113(R).

[26] S. Han , E.‐G. Moon , Phys. Rev. B 2018, 97, 241101(R).

[27] S. V. Syzranov , B. Skinner , Phys. Rev. B 2017, 96, 161105(R).

[28] W. Chen , H. Z. Lu , O. Zilberberg , Phys. Rev. Lett. 2019, 122, 196603. 31144913 10.1103/PhysRevLett.122.196603

[29] Y. Shao , A. N. Rudenko , J. Hu , Z. Sun , Y. Zhu , S. Moon , A. J. Millis , S. Yuan , A. I. Lichtenstein , D. Smirnov , Z. Q. Mao , M. I. Katsnelson , D. N. Basov , Nat. Phys. 2020, 16, 636.

[30] Q. Xu , R. Yu , Z. Fang , X. Dai , H. Weng , Phys. Rev. B 2017, 95, 045136.

[31] S. Li , Z. Guo , D. Fu , X. C. Pan , J. Wang , K. Ran , S. Bao , Z. Ma , Z. Cai , R. Wang , R. Yu , J. Sun , F. Song , J. Wen , Sci. Bull. 2018, 63, 535. 10.1016/j.scib.2018.04.011 36658839

[32] S. Jeon , M. Oh , Curr. Appl. Phys. 2024, 68, 58.

[33] H. Kim , J. M. Ok , S. Cha , B. G. Jang , C. Il Kwon , Y. Kohama , K. Kindo , W. J. Cho , E. S. Choi , Y. J. Jo , W. Kang , J. H. Shim , K. S. Kim , J. S. Kim , Nat. Commun. 2022, 13, 7188. 36418308 10.1038/s41467-022-34845-x PMC9684491

[34] Y. K. Song , G. W. Wang , S. C. Li , W. L. Liu , X. L. Lu , Z. T. Liu , Z. J. Li , J. S. Wen , Z. P. Yin , Z. H. Liu , D. W. Shen , Phys. Rev. Lett. 2020, 124, 056402. 32083898 10.1103/PhysRevLett.124.056402

[35] J. Jeon , J. Jang , H. Kim , T. Park , D. Kim , S. Moon , J. S. Kim , J. H. Shim , H. Min , E. Choi , Phys. Rev. Lett. 2023, 131, 236903. 38134786 10.1103/PhysRevLett.131.236903

[36] Y. Zhao , J. Wyrick , F. D. Natterer , J. F. Rodriguez‐Nieva , C. Lewandowski , K. Watanabe , T. Taniguchi , L. S. Levitov , N. B. Zhitenev , J. A. Stroscio , Science 2015, 348, 672. 25954005 10.1126/science.aaa7469

[37] R. Dombrowski , C. Steinebach , C. Wittneven , M. Morgenstern , R. Wiesendanger , Phys. Rev. B 1999, 59, 8043. 10.1103/PhysRevLett.84.5588 10991001

[38] D. S. L. Abergel , T. Chakraborty , Phys. Rev. Lett. 2009, 102, 056807. 19257539 10.1103/PhysRevLett.102.056807

[39] H. Hadipour , E. Şaşıoğlu , F. Bagherpour , C. Friedrich , S. Blügel , I. Mertig , Phys. Rev. B 2018, 98, 205123.

[40] W. Bauhofer , M. Wittmann , H. G. v. Schnering , J. Phys. Chem. Solids 1981, 42, 687.

[41] P. Blaha , K. Schwarz , G. K. H. Madsen , D. Kvasnicka , J. Luitz , R. Laskowsk , F. Tran , L. Marks , L. Marks , J. Chem. Phys. 2019, 152, 074101. 10.1063/1.5143061 32087668

[42] F. Tran , P. Blaha , Phys. Rev. Lett. 2009, 102, 226401. 19658882 10.1103/PhysRevLett.102.226401

[43] H. Zhang , S.‐C. Zhang , in Tpology and Physics (Eds: C. N. Yang , M.‐L. Ge , and Y.‐H. He ), World Scientific, Singapore 2019, pp. 205–214.

