# Supplementary material for: Direct Observation of Anisotropic Coulomb Interaction in a Topological Nodal Line Semimetal
Source: Adv Sci (Weinh). 2025 Jan 7;12(8):2407437. doi: 10.1002/advs.202407437 (PMC11848639; doi:10.1002/advs.202407437)
Supplement: Supplementary file 1 — Supporting Information [file ADVS-12-2407437-s001.docx]

Supporting Information

**Direct observation of anisotropic Coulomb interaction in a topological nodal line semimetal**

*Hyo Won Kim, Junseo Jung, Gahee Lee, Taesu Park, Won-Jun Jang, Hoil Kim, Jun Sung Kim, Ji Hoon Shim^*^, Bohm-Jung Yang^*^, Sangjun Jeon^*^*

**Section I. Crystal growth and transport property of SrAs_3_**

SrAs_3_ single crystals were synthesized using the Bridgman method. The stoichiometric mixture of Sr (99.99%) and As (99.99%) pieces was sealed in an evacuated quartz ampoule. The ampoule was heated to 550°C, held at this temperature for 2 hours, heated up slowly to 750°C at a rate of 50°C/day, and annealed for an additional 2 days. After X-ray diffraction (XRD) and energy-dispersive spectroscopy (EDS) characterization, the precursor powder was sealed in a graphitized quartz ampoule and grown using the Bridgman method. The ampoule was heated to 800°C and kept at this temperature for a day to fully melt the precursors, followed by slow movement at 0.5 mm/hour from the hot zone to the cold zone with a 5 K/mm temperature gradient, resulting in cylindrical-rod-shaped crystal with a diameter of approximately 12 mm (**Figure S1a**). High crystallinity and stoichiometry of single crystals are confirmed by XRD and EDS.


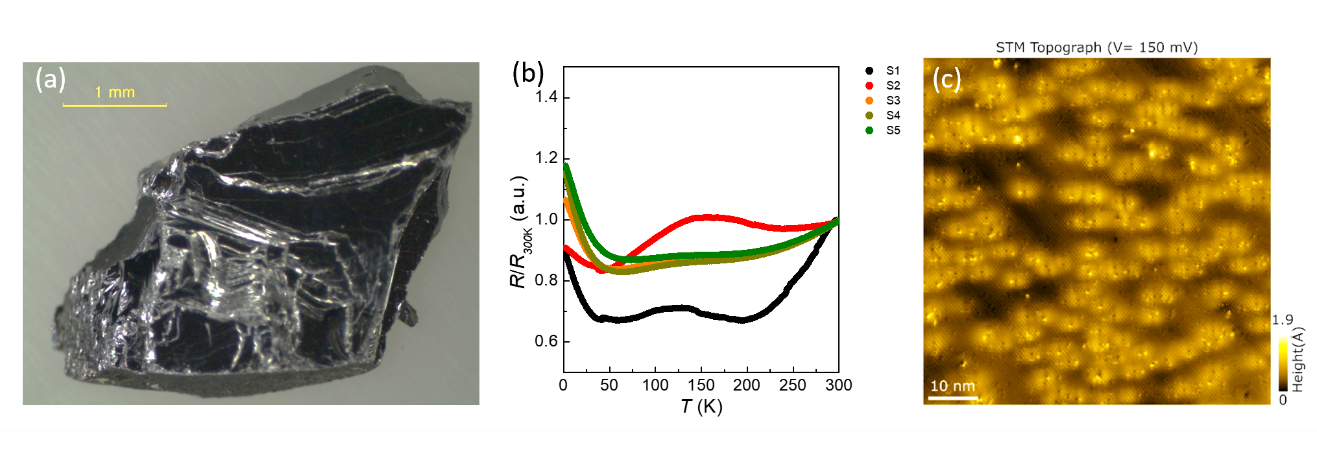


**Figure S1.** a) A picture of a single crystal studied in this study. b) The temperature dependence of resistance. For an STM study, we choose slightly hole-doped single crystals to have stable tunneling current at low energy. c) A typical STM topographic data of the hole-doped single crystals. The bright spots are the charge screening induced by the As deficiency. The density of As deficiency on a cleaved surface is about $4\times{10}^{-12} {cm}^{-2}$.

In our measurements, we selected slightly hole-doped single crystals to ensure stable tunneling currents. The undoped sample exhibited strong tip-induced field effect, hindering low-energy electronic states. While the overall transport properties were insulating at very low temperature as shown in **Figure S1b**, the local averaged density of states (DOS) remains finite near the Fermi level.

**Section II. Energy-dependent charge distribution by an As deficiency**

Energy-dependent scanning tunneling spectroscopy (STS) measurements are carried out around the As vacancy site. The STM probe tip is stabilized with the tunneling condition of V = -125 mV and I = 100 pA at each spatial position. The tunneling conductance (dI/dV) is sequentially obtained by varying the sample bias from -125 mV to +125 mV by using bias modulation of 10 mV. Uniform charging states are visible below -80 mV, butterfly-shaped charge screening is visible between -80 mV and 80 mV, and Friedel-like charge screening is obtained above 100 mV. The diameter of the ring-shaped LDOS enhancement above 100 mV shrinks with increasing sample bias. Additionally, STS measurements are also performed under the same conditions in the impurity-free region, where no charge screening is observed.


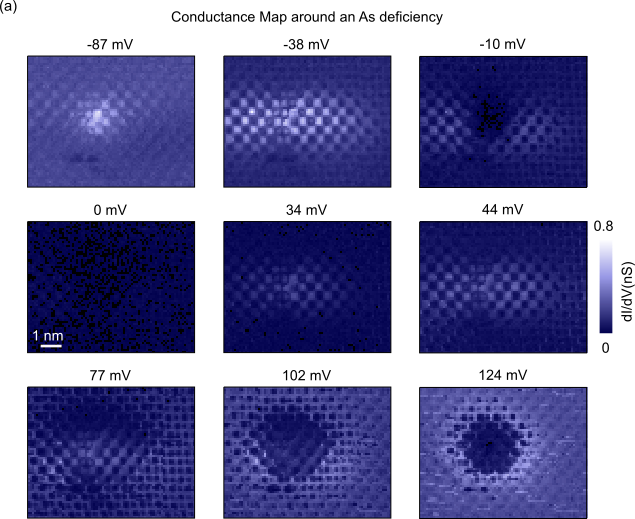


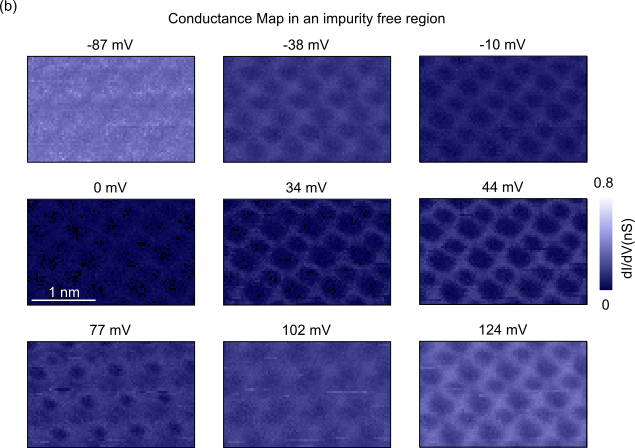


**Figure S2.** a) Energy-dependent STS conductance map around an As deficiency. STS conductance map measured at the indicated bias voltage. The anisotropic charge conductance enhancements are seen from -80 mV to 80 mV. The Friedel-like charge screening is observed above 100 mV. b) Energy-dependent STS conductance map in an impurity-free region. For comparison with (a), the conductance maps are displayed at the same energy as in (a). STM tunnel junction was stabilized with the V = -125 mV and I = 100 pA. The lockin oscillation was 10 mV. The same color scale is used in all maps.

**Section III. Lattice deformation and LDOS variation around an As deficiency.**

High-resolution STM and STS measurements are performed on a defect-free surface of SrAs_3_. The mirror-symmetric As^2^ and As^3^ atoms are clearly imaged with the measurement condition of V = -200 mV and I = 30 pA (**Figure S3a**). The height profile along the $z$ axis reveals the height difference between As^2^ and As^3^ atoms by a spontaneous symmetry breaking (**Figure S3b**). The local DOS (LDOS) of surface atoms shown in **Figure S3c** are plotted in **Figure S3d**. The near-zero tunneling conductance at Fermi-energy is consistent with the semi metallic nature of SrAs_3_.


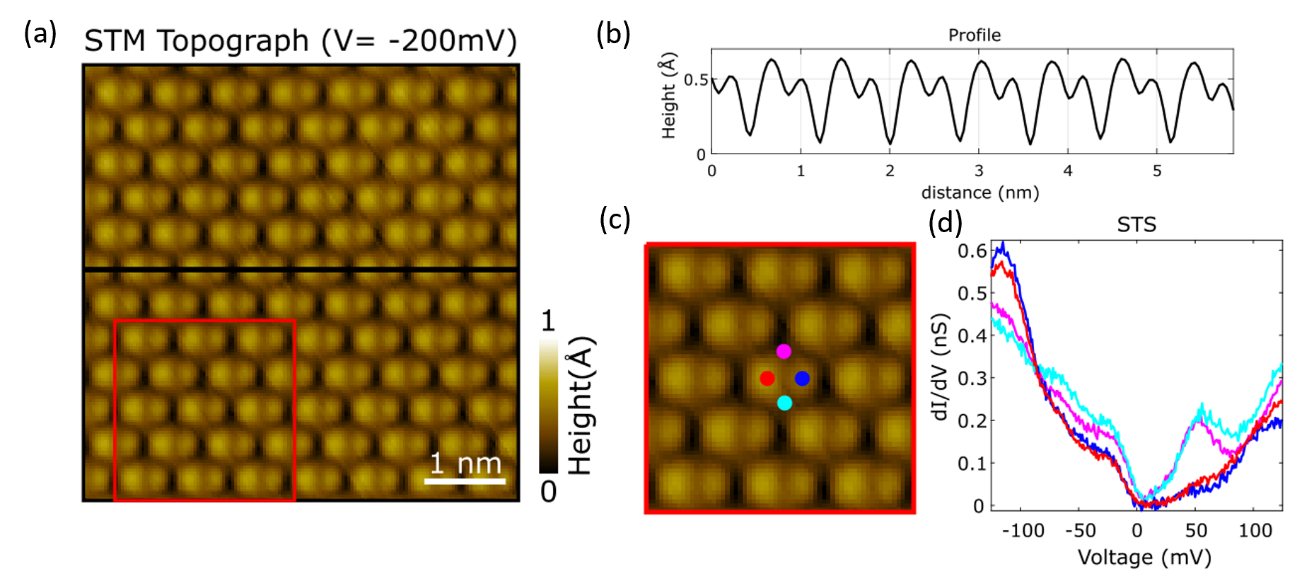


**Figure S3.** a) A typical topographic data obtained on a defect-free surface. b) The height profile along the As-As bonding direction (along with the black line in (a)). The mirror symmetric As^2^ and As^3^ atoms show slightly different heights (~ 10 pm). c) Detailed topographic data of pairs of As atoms. d) The point spectroscopy (dI/dV) marked with colored dots in (c).

The atomic deficiency introduces stronger symmetry breaking near the defect site as displayed in **Figures S4a** and **S4b**. The topographic profiles along the As-As bonding direction are shown in **Figure S4b**. In a regular region, the odd-numbered site is higher than the even-numbered site. In a row containing deficiency site (position 8 of the red profile), the up-down pattern reverses for the 3-4 pair and 5-6 pair. This experimentally obtained broken symmetry and deficiency induced lattice deformation is well captured in DFT calculation (see Supporting Information section IV).

The gap-like feature develops near the impurity site as shown in **Figures S4c** and **S4d**. A pronounced shift in atomic positions, coupled with a significant alteration in LDOS, highlights the robust coupling between electronic states and the lattice in SrAs_3_. The experimentally obtained spatial distribution of lattice deformation is simulated in a DFT calculation (SI section IV) and the anisotropic distribution of charge screening is reproduced in our model calculations (**Figure 4** and SI section V).


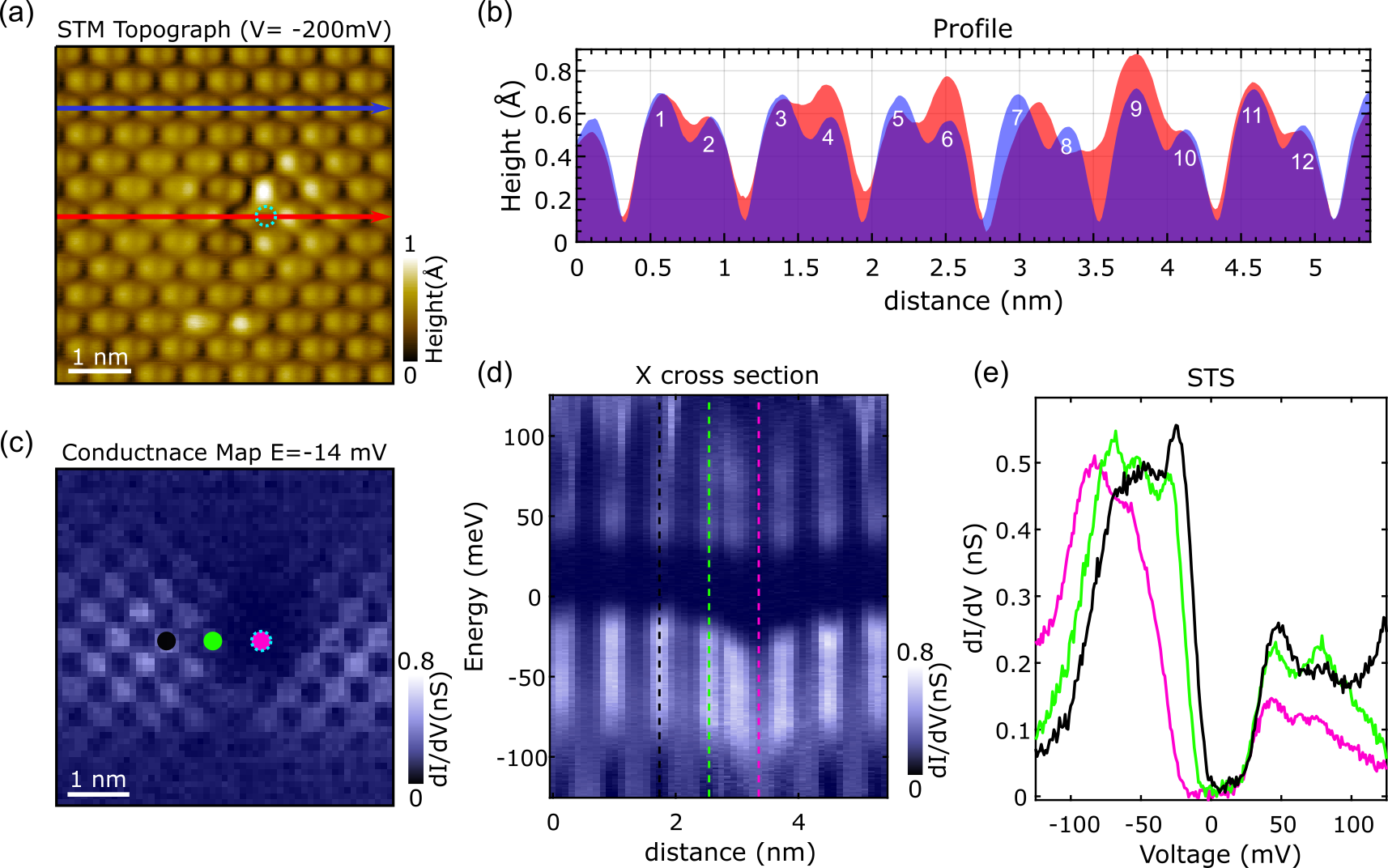


**Figure S4.** a) Typical topographic data obtained near an As deficiency. b) The height profile along the As-As bonding direction. The red curve follows the line through the deficiency (red line in (a)) and the blue curve follows the blue line in (a), 2 nm away from the red line. c) conductance map at -14mV. The red dot indicates the location of the deficiency. d) Continuous STS acquired along the line through the deficiency site. Conductance suppression near Fermi energy is shifted into negative energy at the deficiency site. e) The individual STS curve at the marked position in (c).

The dI/dV map reveals that the increased conductance resulting from arsenic deficiency gradually diminishes over a few nanometers. This decay rate varies depending on direction, with a slower decrease observed along the arsenic bonding axis and a relatively faster decrease parallel to the mirror plane. To further analyze this decay rate quantitatively, polynomial curve fitting was conducted. The fitting expression utilized is $a{(r-r_{0})}^{b}+c$. Where $r_{0}$ is the location of the vacancy, $a$ is the scale, $b$ is the exponent, and $c$ is the conductance offset. Due to the disparity in the state between the As^2^ and As^3^ atoms surrounding the vacancy, the conductance exhibits oscillations during the decay. To address this, we fitted the raw data after convolving it with a Gaussian function that has a larger width than the oscillation period. The fitting was performed approximately 1 nm away from the deficiency to capture the tail of decrease. The resulting exponents are -0.1 in the horizontal direction and -1.5 in the vertical direction, indicating that the charge screening falls slower than the expected $1/r^{2}$ parallel to the mirror plane and significantly slower along the As bonding axis. Considering the finite energy shift from the Dirac energy, tilting of Dirac dispersion, and effective radius of the torus-shaped nodal ring, the charge screening is less effective along the As bonding axis. We note that because the tunneling conductance measures the change in tunneling ratio due to the charge accumulation near the impurity, the decrease of tunneling conductance in space is not directly proportional to the decrease of charge accumulation.

**
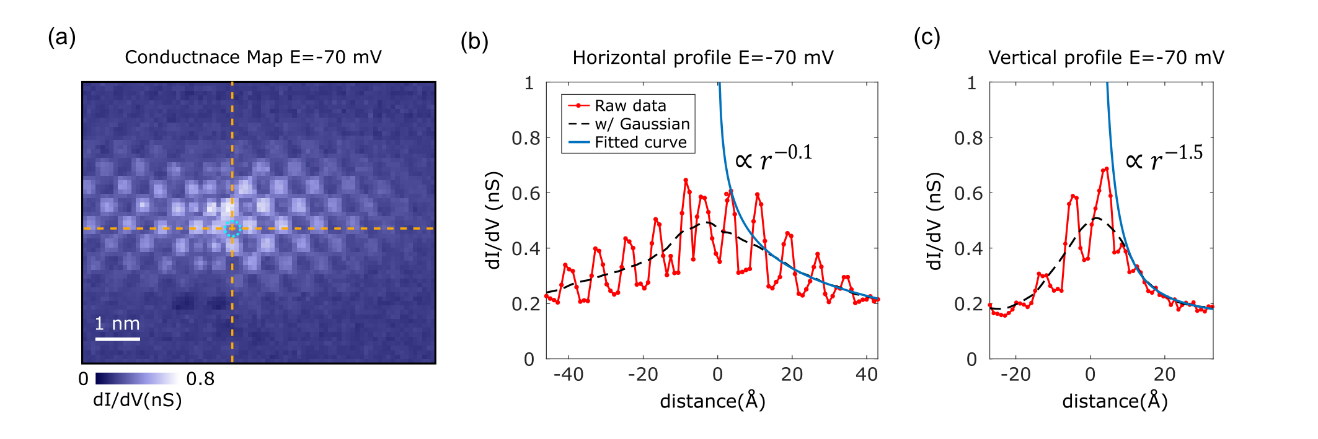
**

**Figure S5.** a) A tunneling conductance measured around an As vacancy. The measurement energy is -70 mV. b,c) The decay of tunneling conductance along the horizontal and vertical direction (red curves). The conductance difference due to the As^2^ and As^3^ atoms results in spatial oscillation in conductance. The polynomial fitting curve (blue curves) fits with the raw data convoluted with the Gaussian window function (dashed black line).

**Section IV. First principle calculation of crystal and electronic structure of SrAs_3_.**

To investigate electronic structure modulation induced by vacancy or impurity on a SrAs_3_ surface, DFT calculation of SrAs_3_ for freestanding slab and surface reconstructed slab was performed. For the slab calculation, we employ the projector-augmented plane wave method implemented in the Vienna ab initio simulation (VASP) package.^[1]^ Firstly, a freestanding defect-free SrAs_3_ surface is constructed using 10 layer slab of SrAs_3_ stacked along c direction with 12 Å vacuum to avoid interactions due to periodic cells, as shown in **Figure S6a**. Sr termination is considered in slab construction as the natural cleavage plane lies between Sr layers. The band structure of surface SrAs_3_ is also calculated for this 10-layer SrAs_3_ slab, confirming the nodal-ring semimetallic feature seen along $\bar{\Gamma}-\bar{T}$ direction as a result of the nodal ring centered at the Y point in the bulk Brillouin zone.


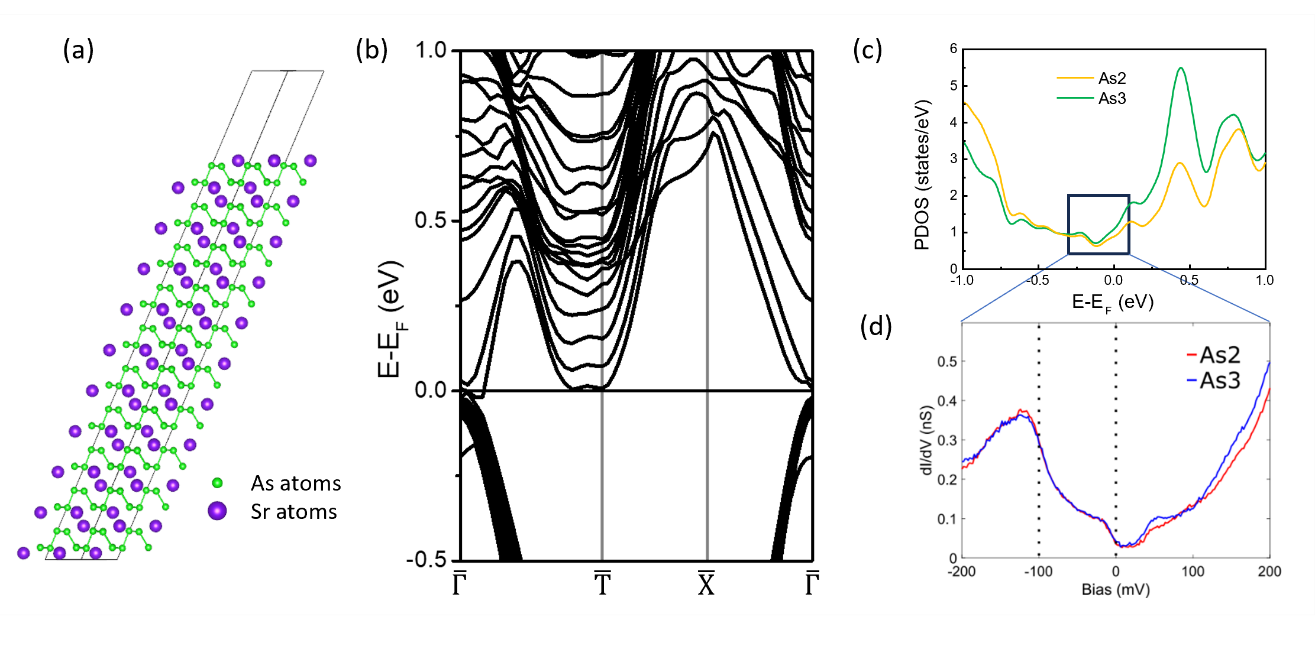


**Figure S6.** DFT calculation of Sr terminated SrAs_3_ slab. a) Constructed crystal structure of defect-free surface SrAs_3_. b) The band structure of the constructed SrAs_3_ slab using mBJ functional. c) Partial density of states for outermost As^2^ and As^3^ atoms in surface reconstructed SrAs_3_ slab where mirror symmetry is broken. d) STS spectra taken at different surface As sites as visualized in **Figure 3**.

In the next step, we simulate the impact of mirror symmetry breaking and long-range effects caused by atomic deficiencies in the SrAs_3_ surface by constructing a $4\times3\times4$ supercell of SrAs_3_ slab, maintaining the same Sr termination and vacuum slab conditions. The internal atomic positions on the top surface are optimized with a force criterion of less than 0.01 eV/ Å. After atomic relaxation, we observe a breakdown in the mirror symmetry of the surface structure within the As chain, resulting in a lower total energy. The partial density of states calculations for the outermost As^2^ and As^3^ atoms in the reconstructed SrAs_3_ slab (**Figure S6c**) properly reproduce the LDOS data obtained from the STS measurements (**Figure S6d**).


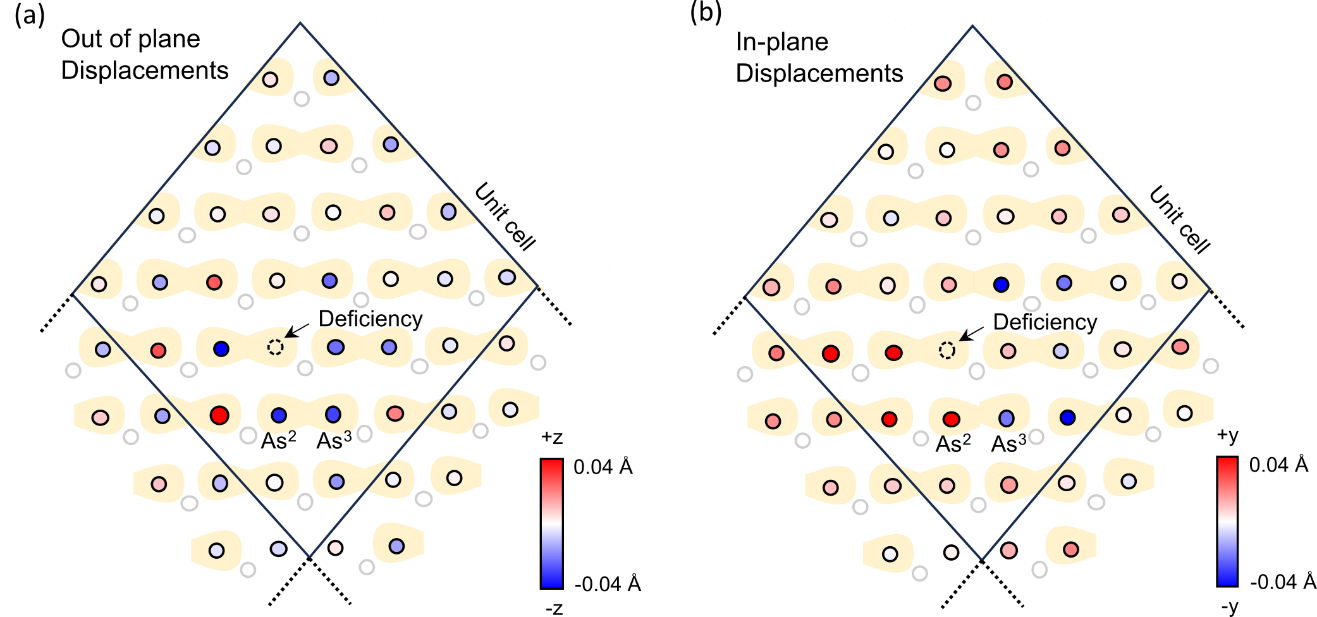


**Figure S7.** Relaxed atomic structure of SrAs_3_ surface with a defect. a,b) Out-of-plane (a) and In-plane (b) atomic displacements of surface As atoms in atoms in 4 x 3 x 4 SrAs_3_ supercell. Atomic positions at the surface are optimized by first-principles calculation. The dashed circle indicates the site of the As vacancy. The total displacements along the As^2^-As^3^ bonding are stronger than the axis parallel to the mirror plane.

In addition, a $4\times3\times4$ supercell of SrAs_3_ with a single As vacancy on top of a surface was simulated by DFT calculation (**Figure S7**). After atomic relaxation, we found strong atomic distortions near the atomic vacancy on a SrAs_3_ surface. Notably, the position deviation along the As^2^-As^3^ bonding is stronger than the axis parallel to the mirror plane which is consistent with the experimentally obtained topographic profile and anisotropic charge screening in our model calculations.

To examine the DOS modification by symmetry breaking, we revisited a 10-layer defect-free SrAs_3_ slab, introducing a height difference between surface As^2^ and As^3^ atoms. As shown in **Figure S8**, the DOS at the SrAs_3_ surface was analyzed for various height differences. The stronger symmetry breaking results in a strong deviation of DOS near the Fermi level reproduces the experimentally obtained STS results shown in **Figures 3d** and **S4e**.


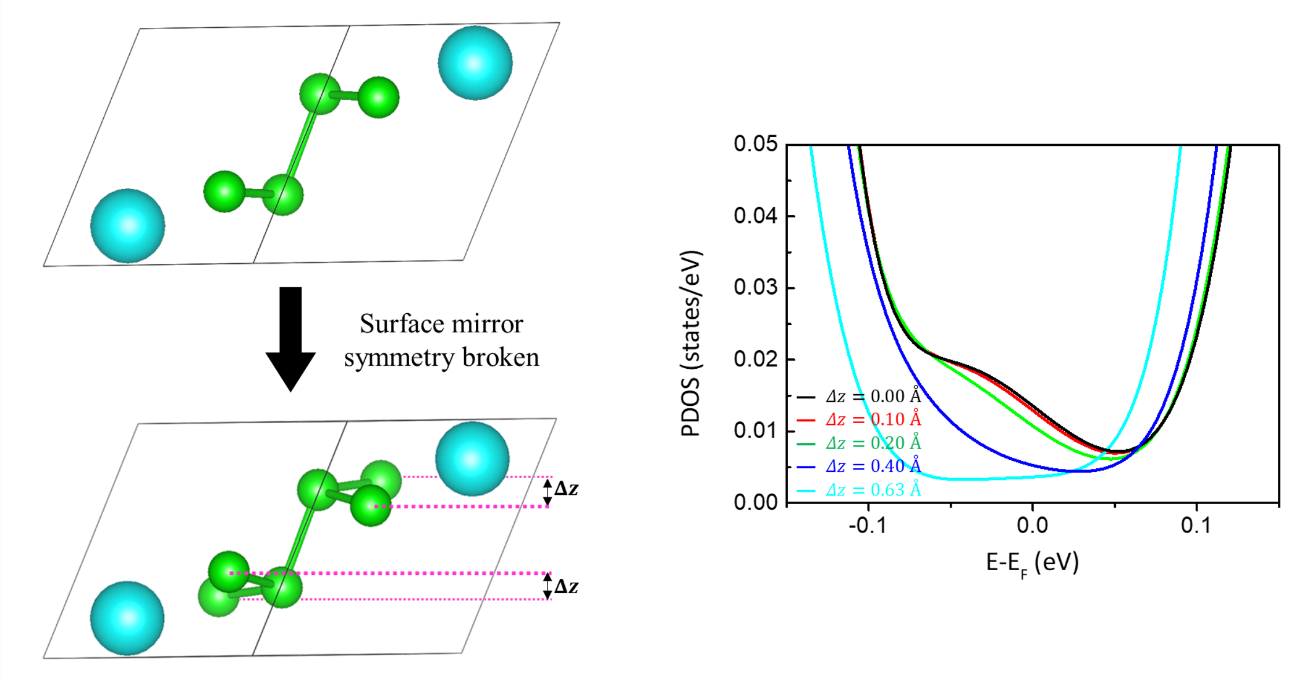


**Figure S8.** Calculated DOS of SrAs_3_ with broken mirror symmetry. Partial density of states as varying surface As^2^-As^3^ height difference from zero to mirror broken defect-free slab realized in DFT calculation for a 10-layer SrAs_3_ slab.

**Section V. Model calculation of charge distribution in a tilted Dirac nodal line semimetal.**

The polarization function of the NLSM is obtained by integrating the polarization function of the tilted Dirac cone [shown in Equation (2) in the main text] along the nodal line. Lindhard formula of the polarization function calculates the density of state difference over the energy difference for a set of points with a constant momentum difference $q.$ For the nodal line, the energy difference between two points with a momentum difference $q$ depends on the direction of the momentum with respect to the nodal line. Thus, when integrating the polarization function, we consider the direction of the momentum with respect to the nodal line position. The polarization function is,

| $Re\Pi^{NLSM}\left( q_{r},q_{z} \right)$ | $=\int_{0}^{2\pi} \Pi^{Dirac}\left( q_{r}\cos\theta,q_{z} \right)d\theta$ |
| --- | --- |
|  | $=-\int_{0}^{2\pi} \frac{q_{r}\cos^{2} \theta+\gamma^{-2}q_{z}^{2}}{\sqrt{\left( 1-\eta^{2} \right)q_{r}^{2}\cos^{2} \theta+\gamma^{-2}q_{z}^{2}}}d\theta$ |
|  | $=-\int_{0}^{2\pi} \frac{q_{r}^{2}+\frac{1}{\gamma^{2}(1-\eta^{2})}q_{z}^{2}-q_{r}^{2}\sin^{2} \theta-\frac{\eta^{2}}{\gamma^{2}(1-\eta^{2})}q_{z}^{2}}{\sqrt{\left( 1-\eta^{2} \right)q_{r}^{2}\cos^{2} \theta+\gamma^{-2}q_{z}^{2}}}d\theta$ |
|  | $=-\frac{1}{1-\eta^{2}}\int_{0}^{2\pi} \sqrt{\left( 1-\eta^{2} \right)q_{r}^{2}\cos^{2} \theta+\gamma^{-2}q_{z}^{2}}d\theta$  $+ \frac{\eta^{2}q_{z}^{2}}{\gamma^{2}(1-\eta^{2})}\int_{0}^{2\pi} \frac{1}{\sqrt{\left( 1-\eta^{2} \right)q_{r}^{2}\cos^{2} \theta+\gamma^{-2}q_{z}^{2}}}d\theta$ |
|  | $=-\frac{\sqrt{\left( 1-\eta^{2} \right)q_{r}^{2}+\gamma^{-2}q_{z}^{2}}}{1-\eta^{2}}E\left( \sqrt{\frac{\left( 1-\eta^{2} \right)q_{r}^{2}}{\left( 1-\eta^{2} \right)q_{r}^{2}+\gamma^{-2}q_{z}^{2}}} \right)$  $+ \frac{\eta^{2}q_{z}^{2}}{\left( 1-\eta^{2} \right)\gamma^{2}\sqrt{\left( 1-\eta^{2} \right)q_{r}^{2}+\gamma^{-2}q_{z}^{2}}}K(\sqrt{\frac{\left( 1-\eta^{2} \right)q_{r}^{2}}{\left( 1-\eta^{2} \right)q_{r}^{2}+\gamma^{-2}q_{z}^{2}}}),$ |
|  |  |

where we obtain the full polarization formula of the nodal line. To obtain the polarization function of SrAs_3_, we use the DFT calculation to extract the tilt and anisotropy strength at the four points on the nodal line. For the representative value of $\eta$ and $\gamma$, we take the average velocity of $v_{r}, v_{z}, v_{r0}$ of all directions and calculate the average tilt and anisotropy strength as shown in **Figure S9**. We substitute these values in the polarization function of the NLSM to obtain the screening charge of the SrAs_3_ (**Figure S10**).


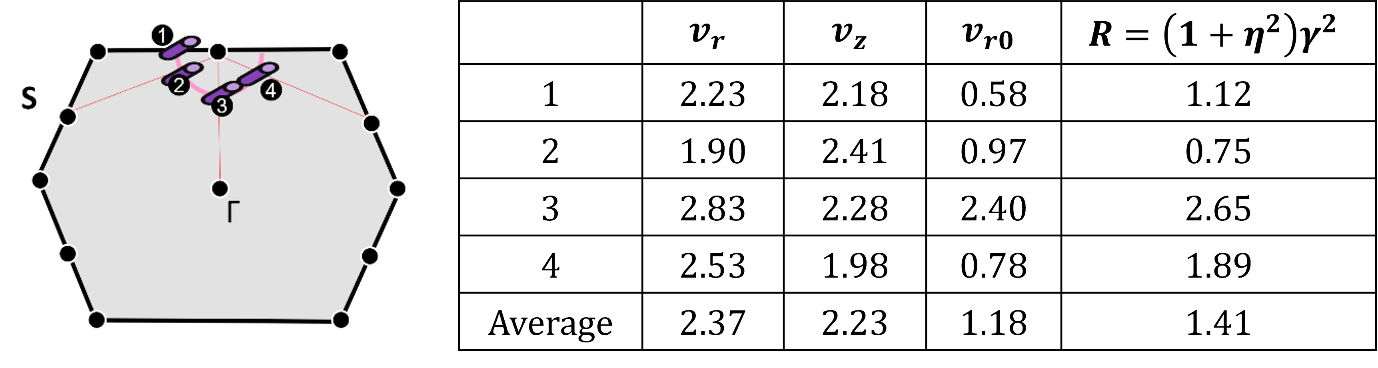


**Figure S9.** Anisotropy values at different points of a nodal line. Each point of the nodal line has a tilted anisotropic Dirac dispersion. We calculate the velocity anisotropy and tilting strength at four points of the nodal line using DFT calculations. The total anisotropy ratio $R=\left( 1+\eta^{2} \right)\gamma^{2}$ is defined by the velocity anisotropy, $\gamma$, and the tilting strength, $\eta$.

**
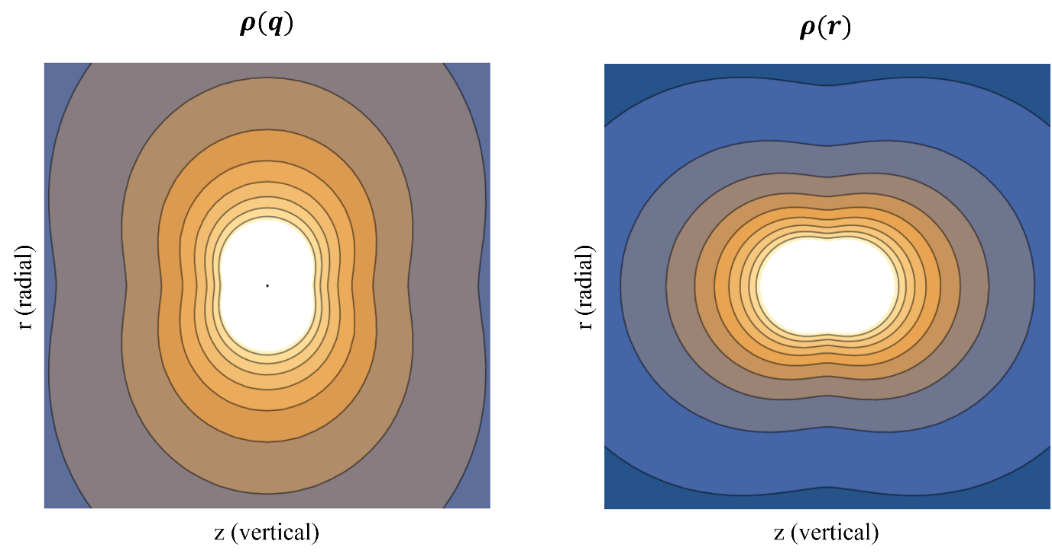
**

**Figure S10.** Anisotropic charge distribution of Dirac nodal line semimetal (DNLS) Screening charge density contour of the nodal line semimetal in momentum space (left) and real space (right). The butterfly shape is shown both in the momentum and in the real space. The average tilt and anisotropy ratio $\eta, \gamma$ which has a value of 0.49, 1.06 respectively, are obtained by averaging the values obtained from several points on the nodal line.


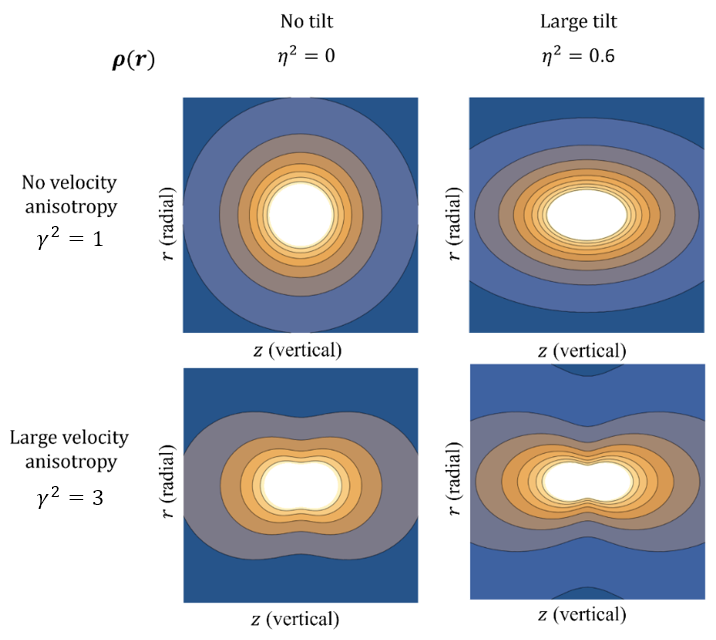


**Figure S11.** Anisotropic charge distribution from tilted Dirac dispersion. As the tilt or the velocity anisotropy increases, the charge density becomes anisotropic, and the butterfly shaped contour appears. The figures show that the contour becomes concave when the condition $\left( 1+\eta^{2} \right)\gamma^{2}>2$ is satisfied.

Next, we explain the sufficient condition for the screening charge to form a butterfly shape. For a tilted Dirac cone, the induced charge density is proportional to the following equation,

$\rho_{ind}\left( q \right)=C\frac{\Pi\left( q \right)}{q^{2}}=C'\frac{q_{r}^{2}+\gamma^{-2}q_{z}^{2}}{(\sqrt{\left( 1-\eta^{2} \right)q_{r}^{2}+\gamma^{-2}q_{z}^{2}})(q_{r}^{2}+q_{z}^{2})}$ .

where $C, C'$ are constants. We assume that the charge density has a longer tail in the radial direction (r) and check whether the contour forms a concave shape. If the contour crosses a point ($0, q_{0})$, then its nearby point is expressed as ($\epsilon, q)$. The contours become concave when $q>q_{0}$. When $\epsilon$ and $q-q_{0}$ are small enough,

| $\frac{1}{{\gamma q}_{0}}$ | $=\frac{\epsilon^{2}+\gamma^{-2}q^{2}}{(\sqrt{\left( 1-\eta^{2} \right)\epsilon^{2}+\gamma^{-2}q^{2}})(\epsilon^{2}+q^{2})},$ |
| --- | --- |
|  | $=\frac{\gamma^{-2}q^{2}}{\gamma^{-1}q*q^{2}}\left( 1+\frac{\gamma^{2}\epsilon^{2}}{q^{2}} \right)\left( 1+\frac{\gamma^{2}\left( 1-\eta^{2} \right)\epsilon^{2}}{q^{2}} \right)^{-\frac{1}{2}}\left( 1+\frac{\epsilon^{2}}{q^{2}} \right)^{-1},$ |
|  | $=\frac{1}{\gamma q}\left( 1+\frac{\gamma^{2}\epsilon^{2}}{q^{2}} \right)\left( 1-\frac{\gamma^{2}\left( 1-\eta^{2} \right)\epsilon^{2}}{2q^{2}} \right)\left( 1-\frac{\epsilon^{2}}{q^{2}} \right),$ |
|  | $=\frac{1}{\gamma q}\left( 1+\left( \frac{\left( 1+\eta^{2} \right)\gamma^{2}}{2}-1 \right)\frac{\epsilon^{2}}{q^{2}} \right).$ |
|  |  |

Thus, for $q$to be greater than $q_{0},$ the tilt and anisotropy strength should satisfy the condition $\left( 1+\eta^{2} \right)\gamma^{2}>2$. We verify this result by plotting charge contours with respect to different tilt and anisotropy strength. **Figure S11** shows that the combined ratio of tilt and anisotropy strength needs to satisfy this inequality for the butterfly shape to form.

**References**

[1] G. Kresse, J. Furthmüller, *Phys. Rev. B* **1996**, *54*, 11169.
